# The Pathophysiology and Management of Hemorrhagic Shock in the Polytrauma Patient

**DOI:** 10.3390/jcm10204793

**Published:** 2021-10-19

**Authors:** Alison Fecher, Anthony Stimpson, Lisa Ferrigno, Timothy H. Pohlman

**Affiliations:** 1 Division of Acute Care Surgery, Lutheran Hospital of Indiana, Fort Wayne, IN 46804, USA; amfecher@gmail.com (A.F.); agstimpson@lhn.net (A.S.); 2 Department of Surgery, UCHealth, University of Colorado-Denver, Aurora, CO 80045, USA; lisalouferrigno@yahoo.com; 3 Surgery Section, Woodlawn Hospital, Rochester, IN 46975, USA

**Keywords:** polytrauma, hemorrhage, shock, resuscitation, coagulopathy, oxygen transport, endotheliopathy, microcirculation, macrocirculation

## Abstract

The recognition and management of life-threatening hemorrhage in the polytrauma patient poses several challenges to prehospital rescue personnel and hospital providers. First, identification of acute blood loss and the magnitude of lost volume after torso injury may not be readily apparent in the field. Because of the expression of highly effective physiological mechanisms that compensate for a sudden decrease in circulatory volume, a polytrauma patient with a significant blood loss may appear normal during examination by first responders. Consequently, for every polytrauma victim with a significant mechanism of injury we assume substantial blood loss has occurred and life-threatening hemorrhage is progressing until we can prove the contrary. Second, a decision to begin damage control resuscitation (DCR), a costly, highly complex, and potentially dangerous intervention must often be reached with little time and without sufficient clinical information about the intended recipient. Whether to begin DCR in the prehospital phase remains controversial. Furthermore, DCR executed imperfectly has the potential to worsen serious derangements including acidosis, coagulopathy, and profound homeostatic imbalances that DCR is designed to correct. Additionally, transfusion of large amounts of homologous blood during DCR potentially disrupts immune and inflammatory systems, which may induce severe systemic autoinflammatory disease in the aftermath of DCR. Third, controversy remains over the composition of components that are transfused during DCR. For practical reasons, unmatched liquid plasma or freeze-dried plasma is transfused now more commonly than ABO-matched fresh frozen plasma. Low-titer type O whole blood may prove safer than red cell components, although maintaining an inventory of whole blood for possible massive transfusion during DCR creates significant challenges for blood banks. Lastly, as the primary principle of management of life-threatening hemorrhage is surgical or angiographic control of bleeding, DCR must not eclipse these definitive interventions.

## 1. Introduction

For the polytrauma patient, brain injury is the most common cause of early death followed by acute blood loss as the second most common cause of early death [1,2]. In the U.S., 150,000 people die each year due to injury and many of these deaths occur in relatively younger individuals, which causes an aggregate loss of productive life of over 3.3 million years [3]. This results in an annual cost to society of USD 2.34 billion in today’s dollars from lost wages and medical costs. In prospective studies that examine resuscitation after trauma the median time to hemorrhagic death is 2.0 to 2.6 h [4,5,6,7]. Hemorrhage is the most common cause of shock in the injured, and a substantial number of trauma patients will arrive at hospital with profound physiologic disturbances due to acute circulatory failure. Dr. Samuel D Gross, regarded as one of the most innovative and influential surgeons of the 19th century described shock simply as, “… a rude unhinging of the machinery of life”. Indeed, this remarkable characterization of hemorrhagic shock remains as informative today as certainly it was over 175 years ago [8].

The polytrauma victim with significant hemorrhage suffers a life-threatening acute reduction in oxygen delivery (DO_2_) to tissue. DO_2_ depends on both an adequate circulating blood volume representing sufficient oxygen carrying capacity, and effective cardiovascular function to maintain the circulation of blood to capillary beds in the periphery.

Furthermore, between 25% to 35% of hemorrhaging patients will develop a biochemically evident coagulopathy (trauma-induced coagulopathy; TIC) before arrival to the emergency department, which can manifest clinically as either hypercoagulable or hypocoagulable states, or both. In the polytrauma patient the presence of TIC is associated with higher transfusion requirements, increased I.C.U. and hospital length of stay (LOS), prolonged requirement for mechanical ventilation, an increase in the incidence of multiorgan dysfunction, and, most concerning of all, a threefold to fourfold higher rate of mortality [9,10,11,12,13]. TIC has deleterious effects independent of injury severity, level of shock, degree of acidosis or depth of hypothermia [14].

Here we examine important pathophysiologic concepts of hemorrhagic shock, and we describe resuscitation strategies for the patient with acute, life-threatening blood loss. Detailed explanations of the complex molecular and cellular aspects of shock and trauma exceed the scope of this review. However, specific advances toward a more complete understanding of hemorrhagic shock at these basic levels may significantly alter future clinical approaches to the polytrauma patient.

## 2. Pathophysiology of Hemorrhagic Sock

### Oxygen Delivery/Utilization Imbalance

The pathophysiology of hemorrhagic shock involves a decrease in systemic DO_2_ to a level less than what is needed to maintain cellular function (VO_2_). DO_2_ equals the rate of blood flow, which is cardiac output (Q; normal = 5–6 L/min) multiplied by the content of oxygen bound to hemoglobin (Hgb) in a volume of blood (normal: male = 20.7 mL O_2_/dL; female = 18.4 mL O_2_/dL). A normal DO_2_ is approximately 1000 to 1250 mL O_2_/min in males, and approximately 925 to 1100 mL O_2_/min for females. If oxygen delivery is insufficient, tissue hypoxia develops resulting in anaerobic metabolism and production of lactate.

**Figure 1:**
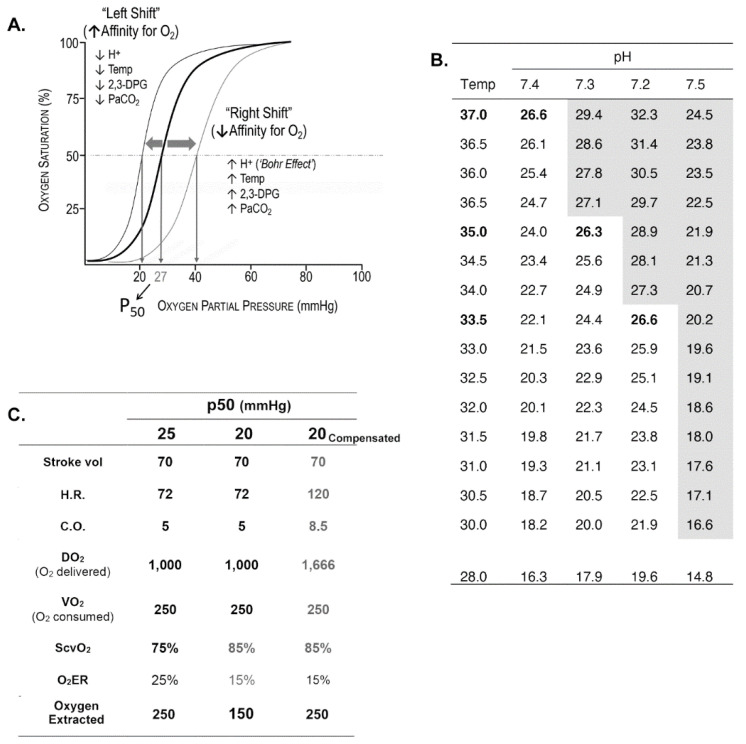
(**A**) OHD curve which relates the saturation of Hgb (*y*-axis) to the degree of partial pressure of oxygen to which Hgb is exposed (*x*-axis). The pO_2_ that saturates ½ of Hgb is referred to as p50, which in this example p50 = 27 mmHg. The p50 is the conventional measure of affinity of Hgb for oxygen. The lower the p50 the higher the affinity of Hgb for oxygen. The ‘steep’ portion of the oxyHgb dissociation curve is in the range of pO_2_ that exists in systemic capillaries (thus a small decrease in systemic capillary pO_2_ can result in the release of large amounts of oxygen for diffusion to, and uptake by cells). As shown in the figure, several factors increase the affinity of Hgb for oxygen (leftward shift; ↓p50) or decrease affinity (rightward shift; ↑p50). Biochemically, H+ is a heterotropic allosteric inhibitor of Hgb, whereas O_2_ is a homeotropic allosteric activator of Hgb. (**B**) Hypothermia and acidosis have opposing effects on p50. Lower temperature shifts the curve to the left increasing Hgb affinity for oxygen and decreasing offloading in capillaries; low pH (increase in H+) decreases the affinity of Hgb for oxygen (Bohr effect) increasing oxygen availability to reverse anaerobic metabolism. A trauma patient may be, and often is hypothermic and acidotic (and coagulopathic). Whether there is a significant change in p50 can be calculated using the Hill–Langmuir equation. (**C**): Hypothetical oxygen transport variables of a normal subject (Temp = 37 °C; p50 = 25 mmHg) and a subject with hypothermia (Temp = 31 °C; p50 = 20 mmHg), before and after compensation. The p50 at 31 °C and pH = 7.4 is calculated using the Hill–Langmuir equation. A venous blood gas is obtained through a Swan Catheter introducer (7.5Fr) with the tip in the superior vena cava reveals in the hypothermic subject, central venous oxygen saturation (ScvO_2_) = 85%. This reflects the fact that hypothermia increases the affinity of Hgb for oxygen, shifting the Hgb dissociation curve to the left. A ScvO_2_ of 85% would imply only 15% of the delivered 1000 mL of oxygen (DO_2_) prior to compensation is being offloaded, which is approximately 150 mL/min, well below VO_2_ (250 mL/min). The hypothermic patient can compensate by increasing cardiac output and hence DO_2_. Assume that stroke volume is unchanged (although a well-known consequence of tachycardia is a reduction in stroke volume), and cardiac output increases by an increase in heart rate (HR) from 72 beats/min to 120 beats/min (a 40% increase in HR causing a substantial increase in myocardial oxygen demand).

An important variable in oxygen transport physiology not often considered because it is seldom measured is the oxygen binding affinity of Hgb, expressed as p50 and depicted by oxy-hemoglobin dissociation (OHD) curves (Figure 1A–C). This property of Hgb determines the extent of peripheral oxygen offloading and therefore the quantity of oxygen available for tissue oxygenation. Acidosis shifts the OHD curve to the right (referred to as the Bohr effect) and increases the offloading of oxygen. Conversely, hypothermia shifts the curve to the left tends to decrease offloading of oxygen in the periphery. Acidosis and hypothermia are frequent homeostatic disturbances that complicate resuscitation. Depending on the magnitude of either one at any one moment during resuscitation, offloading of oxygen from Hgb may be enhanced or impeded [15]. These considerations may explain in part variability of responses to resuscitation of different patients. Additionally, of interest is the possibility of enhancing end-organ oxygen availability in patients with compromised oxygen transport by a pharmacological increase in p50 [16].

Systemic oxygen utilization (VO_2_), approximately 250 mL O_2_/min, is the amount of oxygen consumed each minute by all metabolic processes in the body. The physiologic relationship of VO_2_ to DO_2_ is expressed as the oxygen extraction ratio (O_2_ER),

$$O_{2}\mathrm{ER}=\frac{{\mathrm{VO}}_{2}}{{\mathrm{DO}}_{2}}$$

VO_2_ and thus O_2_ER differ significantly among different organ systems. For example, extraction ratios measured in the in the heart, liver, and kidney, are 60%, 45% and 15% respectively. Predictably, a higher O_2_ER is associated with greater DO_2_ dependency.

O_2_ER provides an important compensatory mechanism offsetting reductions in DO_2_ due to acute blood loss and a decrease in cardiac output. An initial reduction in DO_2_ is offset by an increase in O_2_ER that maintains VO_2_ constant. In this hemodynamic state, the value of VO_2_ is flow-independent. As a compensatory mechanism for blood volume loss, O_2_ER-mediated flow-independence of VO_2_ may result in a deceptive clinical presentation of hemodynamic stability (compensated hemorrhagic shock)*,* although as much as 30 percent of blood volume may have been lost. As cardiac output and thus DO_2_ continue to decline with ongoing hemorrhage, O_2_ER will increase until eventually the amount of oxygen that can be extracted plateaus (O_2_ER = 60–70% for most tissues). From this point, any further decrease in DO_2_ will cause VO_2_ to decline such that the value of VO_2_ is now flow-dependent. The value of DO_2_ that represents the boundary between flow-independent VO_2_ and flow-dependent VO_2_ is designated DO_2 CRIT_. Any DO_2_ < DO_2 CRIT_ is associated with a decrease in VO_2_ and impaired oxygen-dependent cellular processes as metabolism shifts from aerobic to anaerobic pathways.

**Figure 2:**
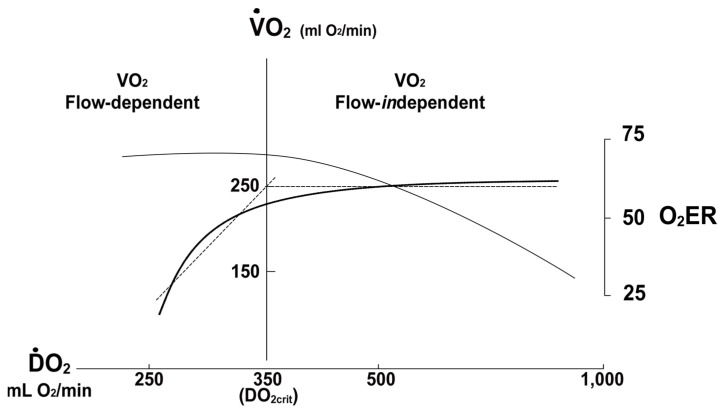
DO_2 CRIT_ defines shock. As DO_2_ (solid black line) decreases secondary to a fall in cardiac output, drop in Hgb concentration, or both, O_2_ER (solid grey line) increases to maintain VO_2_ constant until extraction is maximized. At this point, designated as DO_2 CRIT_ (also referred to as the anaerobic threshold), VO_2_ begins to decrease with further decreases in DO_2_. When DO_2_ > DO_2 CRIT_ t, VO_2_ is flow-independent; when DO_2_ < DO_2 CRIT_, VO_2_ becomes flow-dependent. In addition, DO_2 CRIT_ is associated with the onset of lactate formation and accumulation. Thus, shock can be defined conceptually as the presence of DO_2_ less than DO_2 CRIT_, producing a reduction in VO_2_. Normal DO_2_ = 800 mL O_2_/min/m^2^; normal VO_2_ = 200 mL O_2_/min/m^2^; normal O_2_ER = 25%.

DO_2 CRIT_marks the onset of lactic acidosis and the beginning of an accumulating oxygen debt [17] (Figure 2). Without effective resuscitation, ongoing hemorrhage progresses to decompensated shock, characterized by hemodynamic instability and diminished blood flow that cannot maintain life-sustaining physiologic processes; and then to refractory shock, representing exhaustion of physiological reserves, hemodynamic collapse, vital organ dysfunction and subsequent failure, and ultimately, death.

Therefore, a principal objective of care for the polytrauma patient in shock is to restore DO_2_ to a level (DO_2_ ≈ 350–450 mL O_2_/min/m^2^) such that, to a first approximation, DO_2_ > DO_2 CRIT_. However, targeting even higher, supranormal values for DO_2_ (DO_2_ > 600 mL O_2_/min/m^2^) with aggressive fluid administration predisposes to secondary complications of volume overload. Higher values of DO_2_ likely will not improve survival and, in fact, is associated with detrimental patient outcomes [18].

DO_2_ can be determined from the Hgb concentration, SaO_2_ and stroke volume (hence, cardiac output). Stroke volume can be obtained non-invasively, expeditiously, and to a reasonable degree of accuracy [19] by transthoracic echocardiographic measurement of blood flow velocity at the left ventricular outflow track [20,21,22]. VO_2_ can be estimated as 125 mL/min/m^2^ × BSA (BSA m^2^ = 0.007184 × (W)^0.425^ kg × (H)^0.725^ cm), determined by indirect calorimetry, or calculated using the Fick equation [23]. However, DO_2 CRIT_ is not an exact transition point from flow-independent to flow-dependent VO_2_ [24] and varies considerably from one organ system to another. Moreover, direct point-of-care measurement of many critical parameters of oxygen transport generally are neither practical, nor feasible during resuscitation. Nevertheless, we believe familiarity with the physiology of oxygen delivery/utilization balance, and an appreciation for the meaning of O_2_ER and DO_2 CRIT_, establishes an important conceptual foundation that informs critical decisions typically required during resuscitation.

## 3. Trauma-Induced Coagulopathy

In 2003, Brohi and colleagues identified an acquired coagulopathy in trauma patients recognized as distinct from the coagulation abnormalities caused by dilution during resuscitation [13]. Trauma-induced defects in hemostasis occur in approximately 25 to 65 percent of injured patients who are more likely to be in shock and to have the highest injury severity scores. TIC is associated with increased early transfusion requirements, the development of organ failure, and higher mortality [25]. Mechanistically trauma-induced coagulopathy (TIC), as presently defined, represents a gamut of observed abnormalities in clot formation, fibrinolysis or in any one of several hemostatic pathways that control these two processes.

TIC is associated with diffuse injury to the endothelium (or, endotheliopathy). Trauma-induced endotheliopathy is characterized as a systemic disturbance of microvascular endothelial cell function thought to be caused by exposure to the high levels of circulating catecholamines [26,27,28,29,30,31], and a diverse array of extracellular stimuli, including cytokines such as IL-6 and TNF-α [32]. An important pathologic feature of trauma-induced endotheliopathy is microvascular thrombus formation that blocks flow and oxygen offloading from Hgb in capillary circuits. Shock-induced endotheliopathy occurs together with shedding of the adjacent endothelial glycocalyx. The endothelial glycocalyx is an indistinct layer rich in syndecan-1, hyaluronic acid, heparan sulfate and chondroitin sulfate. The glycocalyx contributes to endothelial cell permeability and function by restricting the movement of fluid and proteins from blood to the interstitium, modulating sheer stress, and controlling inflammatory cell-endothelial cell interactions and associated thrombotic and inflammatory reactions [33]. Shedding of the glycocalyx removes these homeostatic functions and releases proteoglycans that bind and activate endogenous anticoagulant proteins including antithrombin, tissue factor pathway inhibitor (TFPI), and heparin sulfate-like moieties, which essentially heparinize the bleeding trauma patient [34].

TIC also includes fibrinogen depletion and disseminated intravascular coagulation [35,36,37,38,39]. Von Willebrand factor dysfunction may also occur after trauma and is classified as part of TIC. Furthermore, certain qualitative platelet defects develop in trauma patients, particularly in those with head injuries [40,41,42,43,44]. In addition to these coagulopathies trauma patients will demonstrate distinct patterns of dysfunction in the fibrinolytic system ranging from fulminant hyperfibrinolysis to fibrinolysis shutdown, with profound implications for resuscitation strategies.

### 3.1. Specific Defects in Hemostasis Induced by Trauma

#### 3.1.1. Upregulated Protein C Expression

Thrombomodulin (TM) is an endothelial cell surface receptor that binds thrombin. Thrombin-TM interactions promote thrombin-mediated activation of soluble vitamin K-dependent protein C, a reaction that is accelerated by binding of protein C to a co-localizing endothelial cell surface receptor, endothelial cell protein C receptor (EPCR). Activated protein C together with protein S (protein S is named after Seattle the city of its discovery [45]) proteolytically degrades coagulation cofactors VIII and V. Consequently, activations of coagulation factors IX and X are suppressed and thrombin generation from prothrombin terminates [46,47]. In addition to down regulating clot formation, activated protein C/protein S promotes clot lysis by blocking an important inhibitor of fibrinolysis, PAI-1 (plasminogen activation inhibitor-1). Although, thrombin when bound to TM increases anticoagulant activity through activation of protein C, thrombin-TM interactions also promote antifibrinolytic activity by thrombin-mediated activation of TAFI (thrombin-activatable fibrinolysis inhibitor). Activated TAFI interferes with plasminogen binding to fibrin clots, which is required for plasminogen conversion to plasmin by plasminogen activators [48]. Additionally, it is noteworthy that TAFI functions in control of inflammatory processes by modulating complement anaphylatoxin C5a activity [49]. Thus, the clinical variability of TIC may be related in part to the development of endotheliopathy and the countervailing activities induced by upregulated expression of TM.

#### 3.1.2. Von Willebrand Factor

Von Willebrand factor (VWF) is a high-molecular-weight adhesive glycoprotein that plays an essential role in primary hemostasis by promoting platelet adhesion to the subendothelium and platelet plug formation at the sites of vascular injury [50] VWF is increased in plasma and bronchoalveolar lavage (BAL) fluid of patients with acute injury and is predictive of the development of acute respiratory distress syndrome [51]. VWF stored in endothelial cell Weibel–Palade bodies and platelet α-granules after being synthesized in both cell types. VWF ultra-large multimers (ULVWFs) are released from endothelial cells following trauma possibly through systemic endothelial cell activation by IL-1, IL-8, and TNF-α [52]. ULVWFs are then rapidly cleaved to active units by circulating ADAMTS13. Dysregulation of VWF/ADAMTS13 is hypothesized to have a role in propagation of shock-induced endotheliopathy, coagulopathy, and systemic auto-inflammatory reactions. However, despite reports on clinical association between dysregulation of VWF/ADAMTS-13 and poor outcomes of patients with severe trauma, this phenomenon has not been explained mechanistically.

Hypothermia affects all aspects of hemostasis including both procoagulant and anticoagulant activities. However, VWF-platelet glycoprotein receptor Ib-IX-V interactions appear to be the most sensitive to lower temperature [53].

#### 3.1.3. Hypofibrinogenemia

Congenital fibrinogen disorders are rare bleeding disorders affecting either the quantity (afibrinogenemia and hypofibrinogenemia) or the quality (dysfibrinogenemia) or both (hypodysfibrinogenemia) of fibrinogen [54]. Acquired hypofibrinogenemia (depending how it is defined) has been reported in up to 40% of hypotensive trauma patients [55,56,57]. In many cases fibrinogen is the first coagulation component to fall to critical levels [58], and the extent of hypofibrinogenemia correlates with injury severity [59]. Fibrinogen functions as the primary substrate for the coagulation cascade and is converted by thrombin to fibrin strands for clot formation. Fibrinogen is important also for platelet aggregation after engaging the platelet membrane receptor, GPIIb/IIIa. Fibrinogen concentrations < 230 mg/dL are associated with an increase in mortality and moderate hypofibrinogenemia is a determinate of early organ failure, negatively correlating with 24-h SOFA (sequential organ failure assessment) scores [60]. Hypofibrinogenemia is more likely observed in patients with severe extremity or pelvic fractures, who are acidotic and experiencing a long delay in transfer to a trauma center. Specific viscoelastic assays permit rapid assessment of the contribution of fibrinogen to clot strength but must be interpreted with caution [61].

#### 3.1.4. Platelet Dysfunction

Injury, and in particular traumatic brain injury (TBI), is associated with acquired platelet dysfunction, present in nearly 30 percent of patients on admission when assessed by impedance aggregometry in response to arachidonic acid, collagen, or thrombin. Decreased platelet responsiveness to ADP secondary to downregulation of platelet P2Y_12_ receptor has also been well-described [41,42,62,63]. P2Y_12_ is a G-protein coupled receptor that binds adenosine diphosphate (ADP) released from platelet dense granules. Consequently down regulation of this receptor or antagonist blockade inhibits ADP-mediated platelet aggregation. P2Y_12_ inhibition correlates with the severity of TBI as well as TBI-related mortality. The median percent inhibition in TBI patients (mean Glasgow Coma Scale score of 11.9) is 86 percent [42]. Additionally, in patients bearing high injury severity scores and presenting with a severe lactic acidosis (base deficit –8 mEq/L or more), ADP-mediated aggregation is nearly completely inhibited (97 percent). The mean platelet count for all these patients with acquired qualitative platelet defects is normal (232 × 103/µL), and thus impedance aggregometry should be performed. The mechanism responsible for P2Y_12_ down-regulation is not clearly defined.

### 3.2. Dysregulation of Fibrinolysis

#### 3.2.1. Hyperfibrinolysis

**Figure 3:**
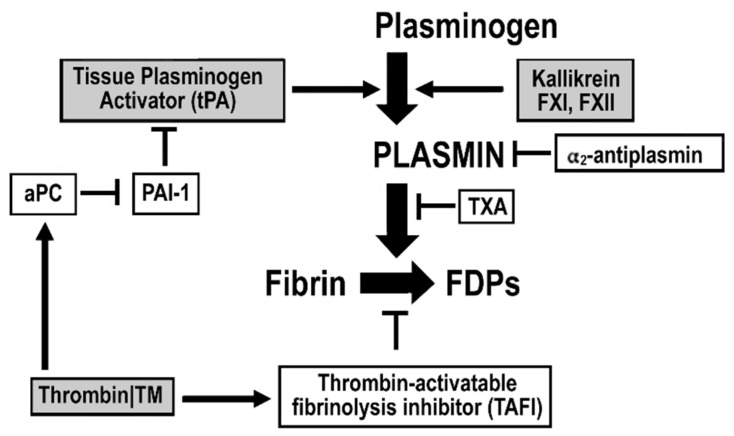
Pathways of plasminogen activation and inhibition. Plasminogen is synthesized by and released from the liver. To be activated to plasmin, plasminogen initially binds to lysine residues exposed on fibrin. The generation of plasmin from its precursor, plasminogen is achieved by the plasminogen activators, tissue-type type plasminogen activator (tPA), and urokinase (not depicted). Protien C, once activated by thrombin bound to thrombomodulin blocks PAI-1, the major inhibitor of tPA; therefore thrombin, through activated protein C, can promote fibrinolysis. However, thrombin-thrombimodulin interactions can also inhibit fibrinolysis through activation of TAFI (thrombin-activatable fibrinolysis inhibitor). Plasmin once formed can also cleave plasma prekallikrein (Fletcher factor) and Hageman factor (FXII) and in turn plasminogen can be activated to plasmin by these proteases. Furthermore, plasmin, can activate the complement factors, C5 and C3, while on the other hand, it can itself be inhibited by the C1-inhibitor, thereby providing a natural means to regulate this process. Excessive plasmin formation can result in hyperfibrinolysis, which increases the risk of bleeding. Tranexamic acid (TXA) blocks lysine-dependent interactions and therefore inhibits binding of plasminogen to and transfusion requirements. Plasminogen receptors located on the surface of immune cells also contain C-terminal lysine the surface of fibrin and misfolded proteins. Plasmin also activates other substrates with pro-inflammatory potential including TGF-β, a neurotrophic agent brain-derived neurotropic factor, and other proteases like the matrix metalloproteinases.

Fibrin has a fundamental role in hemostasis as the product of the coagulation cascade and the principal component in clot formation and as the substrate for fibrinolysis and clot breakdown. Fibrinolysis efficiency is greatly influenced by clot structure, fibrinogen isoforms and polymorphisms, the rate of thrombin generation, the reactivity of thrombin-activated cells such as platelets, and the relative balance of activators and inhibitors of fibrinolysis [64]. Hemostasis is a tightly maintained process that involves formation of clot to arrest bleeding and lysis of clot to maintain vascular patency. Normal clot formation and deposition of fibrin promotes tissue plasminogen activator (tPA)-mediated conversion of plasminogen to plasmin and activation of primary fibrinolysis limiting thrombus growth to the site of injury (Figure 3). tPA is released by fibrin-mediated enhancement of TIC can also be caused by dysregulated fibrinolytic activity [65]. Two major pathologic fibrinolytic patterns are identified in trauma patients: hyperfibrinolysis (HF) and fibrinolysis shutdown (FS). Hemorrhagic shock tends to induce hyperfibrinolysis; tissue destruction, particularly involving solid organs tends to initiate fibrinolysis shutdown [65,66].

HF is a highly lethal, typically fulminant coagulopathy associated with a mortality as high as 75 percent in adults [65] and 100 percent in pediatric patients [67]. This bleeding diathesis develops in approximately 10 to 20 percent of patients who, on admission, will have a higher ISS (>15) and a significantly larger base deficit compared to polytrauma patients without HF. Additionally, hemodilution due to large prehospital crystalloid infusion volumes increase the possibility of patients developing HF compared to patients with similar ISS and base deficit who receive significantly less fluid [68]. Shock-induced endotheliopathy increases TM-mediated activation of protein C. Thus, degradation of the endogenous fibrinolytic inhibitor, PAI-1 by activated protein C results in unregulated accumulation tPA and uncontrolled tPA-mediated induction and amplification of fibrinolysis [69,70,71,72]. This hypothesis has intuitive appeal because it satisfies the principle of parsimony, frequently referred to as the natural law of Occam’s razor, meaning one pathophysiological mechanism (endotheliopathy) links several hemostatic abnormalities that comprise TIC. Conversely it is suggested that lack of detectable PAI-1 activity is not caused by protein C-mediated proteolysis, but rather is secondary to PAI-1 forming covalent complexes with tPA [73].

It is also more apparent, however, from later studies that hyperfibrinolysis is not linked to defects in the coagulation cascade. This conjecture holds that primary HF (and hyperfibrinogenolysis) occurs after a massive shock-induced release of tPA from vascular endothelium. High levels of circulating tPA rapidly sequesterPAI-1 as PAI-1-tPA complexes. tPA in excess of PAI-1 then initiates and propagates systemic fibrinolytic activity by conversion of plasminogen to plasmin. Although other inhibitors and pathways of activation exist for the fibrinolytic system, tPA and PAI-1 interactions predominate [73,74,75,76,77].

#### 3.2.2. Fibrinolysis Shutdown

Whereas HF is the most fulminant form of fibrinolytic dysregulation following severe trauma (ISS ≥ 15), it occurs in in a smaller percentage of severely injured patients (18%) compared to FS, which is observed in 46% of patients. FS is associated with macro-thromboses, resulting in stroke, deep vein thrombosis (DVT), and pulmonary embolism (PE). Additionally, microvascular thromboses can lead to multiple organ failure [78] and eventually death [79]. The mechanism of FS shutdown is thought to be due to massive release of PAI-1. PAI-1 exists in three forms in plasma: (1) free active PAI-1, (2) inactive PAI-1 complexed with t-PA and (3) latent PAI-1 (an inactive PAI-1 conformation). PAI-1 plasma levels vary more than any other component of the fibrinolytic system, likely due to the wide variety of substances that induce PAI-1 production. These include insulin, TNFα, IL-1, transforming growth factor β (TGFβ), and thrombin [80]. PAI-1 is synthesized in hepatocytes and endothelial cells. Platelets α-granules also are a prominent source of PAI-1 (and, α2-antiplasmin) in the circulation after platelet activation with thrombin. However, a mechanistic link between activated platelet PAI-1-mediated inhibition of tPA to fibrinolytic shutdown has yet to be established, whereas platelet dysfunction has been associated with hyperfibrinolysis.

## 4. Management of the Polytrauma Victim

### 4.1. Pre-Hospital Care

#### 4.1.1. Physician-Staffed EMS Response

Twelve percent of trauma deaths may be preventable with advanced resuscitative interventions, which would likely require the presence of physicians in the field or highly trained paramedics [81]. Inclusion of emergency medicine physicians or trauma surgeons in pre-hospital trauma care is, however, controversial. A physician-staffed EMS response to trauma increases the complexity of the care provided at the scene, and this will invariably prolong to some degree scene and total prehospital times. An association between longer scene times and increased mortality in severely injured patients has been demonstrated [82]. Noncompressible bleeding in the abdomen is rapidly fatal, with mortality increasing approximately 1% for each 3 min delay to damage control laparotomy [83]. There are also data to suggest that there is no association between prehospital time and mortality in polytrauma patients [84,85], and that specific patients may benefit more by undergoing advanced airway and chest procedures rather than just faster transport to a trauma center [86]. However, the best prehospital strategies for certain subgroups such as rural trauma patients, patients with multiple blunt force-induced injuries, and perhaps patients undergoing complicated extrications remain unclear [87].

In Germany EMS dispatch is structured on a rendezvous-system between ambulances staffed with paramedics and a vehicle with an emergency physician. The decision to involve the EMS physician is made selectively in an EMS dispatch center. Recently, a telemedicine system was implemented that permits paramedics to consult physicians at anytime. Paramedic-tele-EMS physician consults can bridge the time gap between diagnosis and treatment for patients with life-threatening injuries until the EMS physician arrives at the scene. Furthermore, several potentially life-threatening cases could be handled by a tele-EMS physician as they did not require any invasive interventions that needed to be performed by an onsite EMS physician. Consequently, telemedicine systems establish a higher quality of emergency medical care at an earlier stage [88].

#### 4.1.2. Prehospital Transfusion

Various observations suggest early initiation of resuscitation in the prehospital environment could possibly reduce excessive mortality [89]. To address this issue, two RCTs, the Control of Major Bleeding After Trauma trial (COMBAT (NCT01838863); an individual patient randomized. single-center study design), and the Prehospital Air Medical Plasma trial (PAMPer (NCT01818427); a pragmatic, multicenter, cluster-randomized, phase 3 superiority study design) [90] examined the use of plasma for resuscitation in the prehospital setting. Whereas the COMBAT trial showed that resuscitation with thawed plasma instead of saline for patients in hemorrhagic shock during ground transport (generally with short transport times) did not reduce mortality, the PAMPer trial, in contrast, demonstrated the administration of thawed plasma for hemorrhagic shock during helicopter transport reduced 30-day mortality by 30 percent (23.3% vs. 33.0%; *p* = 0.03) [90]. A post hoc combined analysis of the data from the COMBAT and PAMPer trials revealed that patients who received prehospital plasma transfusion had significantly reduced 28-day mortality compared with standard care, when prehospital transport times were longer than 20 min [91].

Use of whole blood for resuscitation of hemorrhagic shock in the pre-hospital setting has also been examined. A recent study demonstrated that trauma patients who received prehospital LTOWB transfusion had a greater improvement hemodynamically and showed a reduction in early mortality compared to patients who were not transfused, even though the cohort being transfused were in more advanced stages of hemorrhagic shock [92].

#### 4.1.3. Empiric Administration of Tranexamic Acid (TXA)

The Clinical Randomization of an Antifibrinolytic in Significant Haemorrhage-2 (CRASH-2), a pragmatic, randomized, placebo-controlled phase 3 study that involved 274 hospitals in 40 countries, enrolled 20,127 subjects over a five-year period, May 2005 to January 2010, and was funded in part by a major pharmaceutical company that manufacture TXA. The study assessed the effect of TXA on mortality, vascular occlusion events and receipt of blood transfusion following trauma. The study detected a small but statistically significant decrease in 28-day, all-cause mortality deaths of 1.5% in study subjects treated with TXA (1463/10,060 (14.5%) TXA group vs. 1613/10,067 (16.0%) placebo group); death to hemorrhage was reduced 0.8% (489/10,060 (4.9%) vs. 574/10,067 (5.7%)) [93]. In this study of an antifibrinolytic drug, fibrinolytic activity was not measured. Although concerns about CRASH-2 design and methodology persist [94], the results of the study became widely accepted as definitive, and TXA became recognized as the “anti-hemorrhage” drug carried on many ambulances and medical helicopters [95]. In fact, data confirm the effectiveness of TXA when selectively administered to seriously injured patients (mean ISS ≥ 30) during the prehospital phase of care [96,97].

However, in the trauma patient, different states of fibrinolysis other than hyperfibrinolysis can be identified, including inhibition of fibrinolysis and fibrinolysis shutdown representing an inhibition beyond physiologic levels after activation of fibrinolytic pathways [77]. Further inhibition by TXA of a system already demonstrating diminished fibrinolytic activity may increase mortality when given to patients maintaining low but still physiologic levels of fibrinolysis [98], or TXA may precipitate FS in those patients [76]. Thus, inhibition of fibrinolysis in severely injured patients requires careful consideration, recognizing that in certain circumstances TXA can adversely affect survival [65]. Arguably, nonselective administration of TXA to trauma patients is not indicated.

Although TXA is considered primarily an inhibitor of fibrinolysis, it is suggested that early TXA administration also blocks protease-mediated glycocalyx degradation thereby preventing endotheliopathy and associated hemostatic defects [76,99,100].

### 4.2. Hospital Management of the Polytrauma Patient

#### 4.2.1. Initial Assessment

Assessment is commonly based on clinical experience and a set of basic parameters including, level of consciousness, systolic blood pressure (SPB), diastolic blood pressure (DPB), heart rate (HR), respiratory rate, capillary filling time, and capnometry [101]. Hypotension is considered the relevant hemodynamic abnormality in a patient with acute blood loss. However, hypotension is a late finding and suggests physiologic reserves are nearly depleted or have been exhausted. Additionally, hypotension fails to predict the presence of a significant injury or a more immediate requirement for advanced interventions. Shock Index (SI), defined as, HR/SBP may provide a stronger prediction of significant injury [102]. A normal index is essentially < 1.0; thus, whenever the HR is numerically more than the SBP, the patient is in shock. SI ≥ 1.5 is reported to predict massive transfusion for a trauma patient with reasonable sensitivity [103]. A yet more sensitive metric for shock is the modified SI, which is determined by HR/(mean arterial blood pressure); a modified SI ≥ 1.3 indicates a hypodynamic state [104]. The ROPE index is defined as HR/pulse pressure. From the example above, ROPE index = 110/(94−60) = 3.2. This index indicates shock when ≥2.2 and has the potential to be an early indicator of blood loss [105].

For ongoing hemorrhage, the decision to initiate a major resuscitation including massive transfusion is often at the discretion of the trauma surgeon. Twenty-four different scoring systems predict the need for massive transfusion (MT) for a patient with the potential for hemorrhagic shock. Massive transfusion is generally defined as the transfusion of 10 more units of blood within a 24 period; it is also be defined as 3 units of blood per hour (critical administration threshold) [106]. Of scores that use clinical assessment, laboratory values, and ultrasound results, the Modified Traumatic Bleeding Severity Score exhibits the most precision, while the Trauma Associated Severe Hemorrhage score is the most well validated [107]. Recently, a definition of massive transfusion that takes into account the use of whole blood was created that identifies early mortality more accurately than other definitions [108]. Although not widely utilized, noninvasive measurement of muscle oxygenation based on optical spectroscopy may provide the most direct measure of shock and is potentially the best indicator with respect to sensitivity and specificity for massive transfusion [109,110,111,112].

#### 4.2.2. Damage Control Resuscitation (DCR)

Application of evidence-based principles of DCR improves survival in injured patients, although survival of patients with the most severe hemorrhage associated with hypotension is not necessarily improved over older strategies of resuscitation [113]. DCR principles include compressible hemorrhage control; hypotensive resuscitation; avoidance of the overuse of crystalloids and colloids; prevention or correction of acidosis, hypothermia, and hypocalcemia; and hemostatic resuscitation (early use of a balanced amount of red blood cells (RBCs), plasma, and platelets) [114]. DCR can be accomplished using (1) transfusion of whole blood, (2) transfusion of blood components in equal volumes, or (3) transfusion of components directed by results of viscoelastic assay (so-called goal-directed DCR). Notably during DCR microcirculatory function and metabolic cellular function are not measured specifically, directly or continuously in a way that informs decisions in a realistic clinical context. Availability of plasma and platelets is limited in some environments. In these situations, the use of low titer, type O whole blood, thawed or liquid plasma, cold stored platelets or reconstituted freeze-dried plasma can be used as substitutes. Of interest, cold-stored platelets may be superior to room temperature platelets in hemostatic potential [115].

- Resuscitation with whole blood

In 1969, with the advent of component separation of blood at hand, Dr. Francis Moore, former surgeon-in-chief at the Peter Bent Brigham and recipient of the Samuel Gross Medal of the American Surgical Association, published this opinion on resuscitation, “For the restoration of homeostasis after acute massive hemorrhage, it appears that fresh compatible whole blood is the ideal transfusion.” [116]. Restoration of adequate blood volume and correction of trauma-induced defects in hemostasis can be accomplished with transfusion of low titer (anti-A antibodies, anti-B antibodies < 1:256), type O, Rh-negative, whole blood (LTOWB). The use of LTOWB for trauma patients has expanded substantially in U.S level 1 and 2 trauma centers from 2018 to 2020, which includes an increase in the use of Rh-positive LTOWB in females of child-bearing years [117,118]. Benefits of LTOWB-based resuscitation include possibly an increase in survival compared to component-based resuscitation [119,120], reduced donor exposure, all elements critical to hemorrhage control are contained in one product in physiologic amounts [118], and that transfusion of younger red blood cells occurs [119] because of shorter storage times. Length of storage time for whole blood remains debated, although data show significant degradation of the hemostatic potential of whole blood after 14 days of storage [121]. Leukoreduction of LTOWB does not appear to afford any distinct clinical benefit over non-leukoreduced units [122]. Additionally, resuscitation with whole blood may be a better option for exsanguinating hemorrhage in certain parts of the world where there is a lack of well-equipped blood banks and insufficient availability of blood products [123]. However, the percentage of all donors who are eligible to donate RhD-negative LTOWB (male, group O, RhD-negative, and have low titer anti-A and -B) is only 3% RhD-alloimmunization rate is approximately 21% [124].

Successful experience with fresh whole blood by the US military is well documented [125]. Recent studies suggest that LTOWB in resuscitation of civilian trauma is associated with a reduction in post-emergency department transfusions and increase likelihood of 24-h and 28-day survival [120,126]. Conversely, other data suggest that, although safe, resuscitation with LTOWB does not significantly improve survival compare to component-based resuscitation [127,128], and LTOWB does not reduce blood product utilization, as first hypothesized [120]. Arguably, existing studies on LTOWB-based resuscitation are limited for the most part, and a more rigorous, high quality investigation to address the effectiveness of LTOWB may be warranted [117,119,129].

- Fixed Component Ratio-based DCR

The 2015 Pragmatic, Randomized Optimal Platelet and Plasma Ratios (PROPPR) trial established use of balanced blood component transfusions for resuscitation of hemorrhagic shock. This study involving 12 major US. Trauma centers showed resuscitation with blood components transfused in a fixed ratio of 1:1:1 (plasma:PLTs:pRBCs) reduced mortality caused by exsanguination at 24 h when compared to transfusion of components in a fixed ratio of 1:1:2 (9.6% vs. 14.6%) [130]. In both groups, platelets were transfused first followed by plasma alternating with pRBCs. Components transfused 1:1:1 deliver a blood substitute that is anemic, thrombocytopenic and hypocoagulable [131].

Platelets are separated from a unit of fresh whole blood by apheresis or centrifugation. Platelets are customarily stored in the blood bank at room temperature (20–24 °C) for up to 5 days. Platelets stored at this temperature incur a substantial risk of bacterial contamination. Transfusion of platelets stored at room temperature is associated with a greater risk of septicemia and death than transfusion of any other blood product [132]. Moreover, at room temperature, there is rapid deterioration in platelet function referred to as a platelet storage lesion, characterized by, fragmentation, activation, degranulation and aggregation together with increased glycolysis and intracellular acidosis that significantly diminish the efficacy of platelet transfusions [133]. Increased demand for platelets during DCR placed on an always-limited blood bank inventory may significantly challenge resuscitative efforts.

To circumvent these problems, platelet storage at 4 °C is being re-examined [134,135]. Platelets were originally stored at 4 °C until it was shown that the half-life of cold-stored platelets after transfusion was markedly reduced in circulation (1.3 days) compared to the half-life of platelets stored at room temperature (3.9 days) [136]. However, cold stored platelets show evidence of activation including increased thromboxane A2 production and increased surface expression of P-selectin and GPIba receptors [137]. It is suggested that because of pre-activation, cold stored platelets may be more effective than platelets stored at room temperature for hemostatic resuscitation [137]. Cold stored platelets have better adhesion and aggregation functionality than platelets kept at room temperatures, which is associated with a reduction in bleeding times [138]. The issue then becomes whether the short half-life of cold-stored platelets is still sufficient for DCR.

Transfusion of packed red cells at times in massive amounts can reestablish adequate oxygen carrying capacity, although RBC transfusions are associated with measurable risks of morbidity and mortality. After separation from whole blood, pRBC’s are stored at 4 °C in a preservation additive for up to 42 days. Current blood banking procedures may not fully preserve red blood cell (RBC) function during storage, contributing to the decrease of RBC oxygen release ability [139]. The storage time of transfused blood is an independent risk factor for post-injury multiple organ failure [140,141]. During this time RBCs undergo biochemical changes collectively referred to as RBC storage lesion. In addition to developing severe metabolic imbalances, stored RBCs transform morphologically from flexible biconcave discs into rigid shapes (burr cells) that do not readily deform and obstruct flow through capillary beds [142]. Furthermore, microvascular flow is increased by nitric oxide and adenosine triphosphate released from RBCs in hypoxic conditions. Both these microvascular regulatory mediators are depleted in cold-stored RBC’s.

Additionally, cold stored RBCs are depleted of erythrocyte 2,3-diphosphoglycerate (2,3-DPG). Binding of 2,3-DPG to Hgb decreases the oxygen binding affinity of Hgb (rightward shift of the oxyHgb dissociation curve; increase in p50), which facilitates offloading of oxygen from Hgb in tissue. In MT, when a patient may have predominately banked blood circulating, the time needed to synthesize and accumulate 2,3-DPG and regenerate a p50 favorable to tissue oxygenation may be excessive [143]. RBC energy metabolism progressively deteriorates, and energy-dependent redox systems fail. Increasing oxidant stress leads to accumulation of irreversibly oxidized proteins, metabolites, and lipids. Therefore, it is reasonable to consider that a significant red cell storage lesion would negatively affect outcomes in transfused trauma patients, and that fresh blood or blood with relatively a short storage time should be used. Earlier studies in the aggregate are inconclusive regarding whether age of transfused blood has any association with trauma patient outcomes [144]. In one study, an increase in the number of older PRBCs (≥22 days storage) transfused during massive transfusion of trauma patients was shown, in fact, to be independently associated with increased likelihood of 24-h mortality (adjusted odds ratio = 1.05 per PRBC unit; 95% confidence interval) [145]. However, in a more recent analysis, no systematic correlation was found between storage time of transfused RBC units and in-hospital mortality of patients undergoing massive transfusion [146].

Plasma transfusion during DCR provides clotting factors and has been shown to mitigate the endotheliopathy of trauma. Protection of the endothelium may be in part due to fibrinogen and other plasma-derived proteins, although the exact mechanism of endothelial cell protection by plasma has not yet been elucidated [30,31,147]. In the U.S. the designation fresh frozen plasma (FFP) means plasma was separated and frozen within 8 h of collection; plasma produced from whole blood stored at 4 °C for up to 24 h prior to component separation is designated FP24. FFP is rapidly administered early in the course of resuscitation. However, there are technical difficulties with the timely administration of FFP, which is stored at −18 °C and requires 30 to 40 min to thaw, label and issue. Moreover, utilization of FFP is limited by a short half-life once thawed and by the fact FFP from rare blood group AB donors is used before the recipient’s blood type is known. To make plasma readily available at the initiation of DCR, it may be feasible for blood banks to maintain a small inventory of plasma that has been thawed for less than 24 h, which can be issued without delay and transfused immediately while type-specific FFP is thawed.

Thawed plasma or “never-frozen” liquid plasma are two other products that can be used immediately and thereby obviate FFP and FP24 transfusion for management of TIC [148]. Coagulation factor levels are maintained in thawed plasma for up to 5 days with the exception of coagulation cofactor VIII, which falls below the lower limit of normal by the fifth day of storage [149,150]. Additionally, to preserve blood bank inventories of group AB plasma, group A thawed plasma is transfused empirically when the recipient’s blood type is unknown [151,152,153]. The risk of an ABO blood group mismatch reaction is relatively rare since the most common encountered blood group in a recipient will be group A. Moreover, anti-B antibodies generally are low in group A plasma from certain donors, for example, North American males >50 years of age. Greater than 90% of surveyed trauma centers report using type A plasma.

Despite significant declines in some factors in liquid plasma stored for up to 40 days, fibrinogen concentration and clot strength as measured by viscoelastic assay were stable. Liquid plasma is easier to store and prepare and may be more amenable to prehospital transfusion [154].

Freeze-dried plasma (FDP), which can be stored at room temperature for 2 years is another source for clotting factors for transfusion into the hemorrhaging trauma patient. FDP is rapidly reconstituted with sterile water and can be used within minutes. The overall safety and efficacy of FDP may be equivalent to allogenic blood products based largely on observational studies; however, there are no data from larger, randomized controlled trials comparing FDP with FFP to confirm this. Furthermore, the effects of FDP on host auto-inflammatory reactions, if any, are unknown [155]. Finally, concerns regarding disease transmission, including hepatitis, with the use of pooled FDP, led to the cessation of large-scale production [156]. Interest in FDP persists in the military setting, nonetheless, and with significant improvement in donor screening, testing procedures, and pathogen reduction technology, including a photochemical pathogen inactivation process the French military continues to produce French lyophilized plasma (FLyP) [157]. Providing FFP early for civilian patients in the prehospital setting, when TIC is thought to first develop presents formidable technical and logistical challenges. Military applications of FDP suggest, however, the feasibility of plasma transfusion in civilian prehospital and early hospital settings, or in settings where FFP is unavailable [158].

Because hypofibrinogenemia is recognized to significantly complicate hemorrhagic shock, early repletion of fibrinogen [159,160] in concert with platelet, RBC and plasma transfusion is advocated. Several international guidelines for DCR specify fibrinogen infusion for plasma fibrinogen levels <150–200 mg/dL or diminished clot strength due to hypofibrinogenemia (or possibly acquired dysfibrinogenemia [161]) as indicated by viscoelastic assay [162]. In the U.S., cryoprecipitate (cryo) is the principal source of fibrinogen [163], which is produced by slowly thawing FFP. Other coagulation factors enriched in cryo include von Willebrand factor, FVIII, and FXIII, and these plausibly contribute to hemostatic resuscitation. Recent in vitro and in vivo data suggest that cryo potentially attenuates the endotheliopathy induced by hemorrhagic shock [164].

- Goal-directed DCR

**Figure 4:**
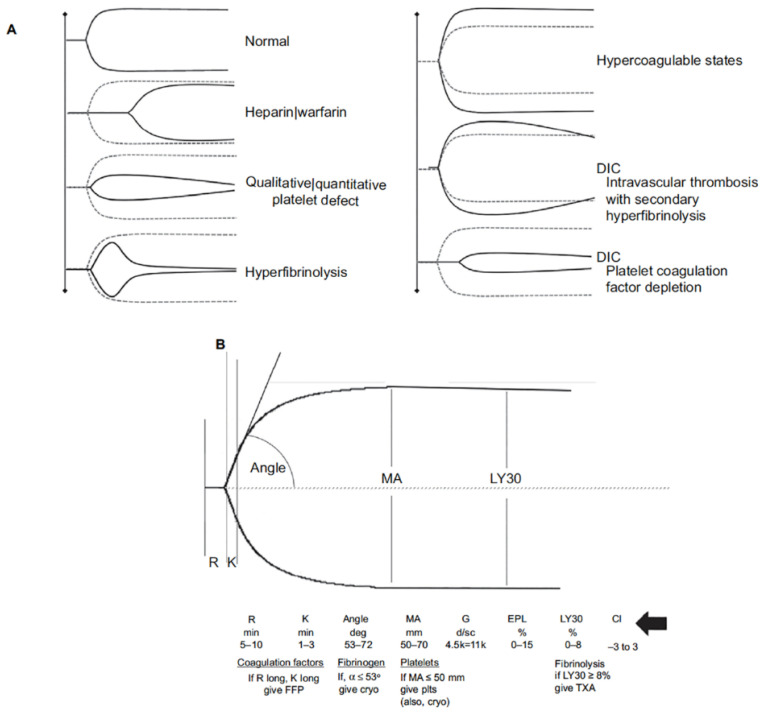
Thromboelastography (TEG^®^). (**A**) Schematic presentation of different viscoelastic tracings reflecting states of the coagulation system compared with normal. (**B**) Basic viscoelastic tracing with measured parameters and limits of normal for thromboelastography, correlated with different elements of the coagulation system (R = reaction time, K = clot formation time, angle, MA = maximum amplitude, Ly30 = percent clot lysis 30 m after MA). Viscoelastic k-time and angle correlate to some degree with fibrinogen concentration. However, the agreement between these parameters and fibrinogen levels determined by standard von Clauss assay is not sufficiently strong to be useful clinically. To overcome this limitation with TEG, the specific contributions of fibrinogen and platelets to clot strength can be determined with additional reagents (TEG; Functional Fibrinogen [Haemonetics Corp, Niles, IL, USA]). Using TEG, additional measures of clot strength can be computed. Coagulation index (CI; black arrow) is derived from R, k-time, angle, and MA, with a CI >+3.0 suggesting a hypercoagulable state and CI <−3.0 suggesting coagulopathy. The shear elastic module strength, designated G, is a computer-generated quantity that reflects an integrated measure of clot strength. Conceptually, G is considered the most informative parameter of clot strength because it reflects the contributions of the enzymatic and platelet components of hemostasis. Abbreviations: rTEG, rapid thromboelastography; DIC, disseminated intravascular coagulation; EPL, estimated percent lysis; FFP, fresh frozen plasma; Cryo, cryoprecipitate; Plts, platelets; TXA, tranexamic acid.

A viscoelastic assay (VEA), by either thromboelastography (TEG) (Figure 4) or rotational thromboelastometry (ROTEM), assesses several parameters of fibrin formation and HF and/or FS in whole blood [165,166,167,168]. VEA-based assessment of coagulation provides a rapid, integrated measure of clot formation and dissolution in blood compared to conventional coagulation tests (CCT) of individual coagulation pathways performed on prepared plasma and therefore in the absence of platelets and red cells. In addition, VEAs can detect hyperfibrinolysis or fibrinolysis shutdown, which are not detectable by CCTs, although on occasion VEAs may fail to detect so-called occult fibrinolysis [169]. Neither VEAs, nor CCTs assess the complex influence of the endothelium on hemostatic activity.

As DCR progresses serial viscoelastic assays on a patient reliably identify particular coagulopathies and delineate fibrinolytic phenotypes within a realistic clinical context and in a timelier fashion than conventional tests of coagulation [170,171]. For example, VEA’s may predict a specific need for plasma or suggest instead cryoprecipitate for TIC even before coagulopathic bleeding is evident clinically. These assays can also predict massive transfusion earlier and more accurately than clinical judgement or CCTs. Viscoelastic assay is particularly useful, for example, in suggesting the possibility of coagulopathy due to platelet dysfunction (MA < 55 on TEG) in a head injury patient with a normal platelet count. Additionally, complicating factors such as the effects of preexisting pharmacological treatment with direct oral anticoagulants can be identified by VEAs.

Viscoelastic assays such as TEG that are used to diagnose hyperfibrinolysis may take as long as 60 min before results are obtained on which to base anti-fibrinolytic therapy with TXA. Delays in TXA administration to trauma patients who are coagulopathic due to hyperfibrinolysis reduce the effectiveness of TXA [172], whereas empiric TXA administration exposes patients without hyperfibrinolysis to risks of TXA-induced VTE and organ failure [98,173]. Of more concern is the potential adverse effects of TXA administration to patients with FS [174]. A complete TEG at POC provides data certainly no sooner that CCTs performed in the laboratory. Rapid TEG accelerates the process by addition of thrombin as a initiating agent, which is accelerated faster still by the addition of plasmin. Added plasmin identifies patients at highest risk for hyperfibrinolysis within 5 min instead of 60 min, and therefore may prove useful for selective and timely administration of TXA [175]. The detection of hyperfibrinolysis can direct selective use of TXA [176] rather than empiric treatment of all trauma patients with this potentially dangerous anti-fibrinolytic. Until recently, VEAs results and the reproducibility of results were dependent on the accuracy of pipetting while performing the assay. This challenge has been obviated and accuracy improved with preloaded cartridge systems with the TEG 6S and ROTEM Sigma [170]. Neither VEAs, nor CCTs include, however, the important contribution of the endothelium to hemostasis [177]. Currently it is suggested that VEAs in DCR may improve survival and reduce blood component transfusion [168,178].

- Resuscitation with Concentrates

Several European countries provide clotting factors as concentrates. Four factor prothrombin complex concentrate (4F-PCC), is a fractionated, heat-treated, nanofiltered, lyophilized, non-activated plasma product made from pooled human donations. 4F-PCC contains the four vitamin–K dependent coagulation factors II, VII, IX and X, and protein C and protein S that also require post-synthesis vitamin K mediated carboxylation of glutamate residues. Protein C and protein S anticoagulant activities balance the procoagulant activity of PCC and reduce the incidence thromboembolic complications associated with this product.

Hypofibrinogenemia and increased fibrinogen breakdown are important findings of TIC and fibrinogen is usually the first factor to reach critically low levels in traumatic hemorrhage [179]. The presence of hypofibrinogenemia on arrival at hospital predicts massive transfusion, and is associated with an increase in morbidity and mortality [180,181,182]. Several studies have examined the feasibility of providing fibrinogen to severely bleeding trauma patients and have suggested that soluble fibrinogen given early during trauma resuscitation may have some clinical benefit [183,184,185,186]. Pre hospital administration of fibrinogen concentrate has been shown to improve clot stability and to prevent significant decreases in plasma fibrinogen. In the U.S., fibrinogen concentrate is used to treat bleeding and for prophylaxis of patients with congenital hypofibrinogenemia [187] but it is not approved for use in patients with acquired disorders of fibrinogen.

Fibrinogen concentrates do not contain platelet membrane microparticles. These small structures are generated in freeze-thaw cycled plasma during preparation of cryoprecipitate and are associated with thrombotic or inflammatory potential in trauma patients [188]. There are several plasma derived fibrinogen concentrates marketed world-wide for the management of acquired hypofibrinogenemia. The manufacturing processes differ suggesting that small but potentially clinically relevant differences in composition may be present including, fibronectin, vWF antigen, vitronectin, albumin, fibrinopeptide A, and plasminogen. Of note, factor XIII is detectable in different products from 0.2 U/mL to 3.9 U/mL [189]. Recent studies demonstrate the combination of added fibrinogen and factor XIII is highly effective in raising maximum clot firmness determined by viscoelastic assay [190]. Factor XIII not only generated stable clot resistance to hyperfibrinolysis but also enhanced platelet function by facilitating clot retraction. High-dose FXIII administration therapy has significant clinical impact for severe trauma. High-dose Factor XIII administration induces effective hemostasis for TIC both in vitro and in rat hemorrhagic shock models [191].

#### 4.2.3. Secondary Assessment

We target intermediate variables of reflecting microcirculatory function such as stroke volume, mean arterial pressure, heart rate, and urine output. Modalities that can be used to monitor microcirculatory dysfunction include determination of the PCO_2_ gap, in-vivo videomicroscopy using orthogonal polarization spectral imaging or sidestream dark field imaging. With NIRS, (Near Infrared Spectroscopy) of oxygenated Hgb in that tissue). The NIRS value of the Hgb oxygen concentration in a tissue is represented as StO_2_ (tissue oxygen saturation) and this value can be obtained for vessels that are less than 1 mm in size.

During resuscitation, reaching satisfactory measures of macrocirculatory oxygen transport do not necessarily indicate adequate perfusion at a microvascular level or sufficient oxygenation of tissue. Additional parameters to assess include serum lactate and the oxygen saturation of central venous blood (ScvO_2_) a surrogate for the saturation of true mixed venous blood (SvO_2_) sampled from the pulmonary artery [192,193,194]. Because there is a lack of agreement between absolute values for SvO_2_ and ScvO_2_, the clinical utility of ScvO_2_ has long been in question [195,196]. However, we believe trends in ScvO_2_ can provide important and accurate information for decision making during resuscitation, although normalization of ScvO_2_ neither excludes persistent tissue hypoperfusion, nor precludes evolution to multi-organ dysfunction and death [197]. The difference between central venous pCO_2_ (pcvCO_2_) and arterial pCO_2_ (paCO_2_), which can be referred to as “pCO_2_ gap” may support deductions regarding oxygen delivery to tissues, although this parameter is applied more frequently to patients in septic shock. A deficit in tissue perfusion secondary to persisting reductions in microvascular flow, or macrovascular flow is considered as a primary cause of a pCO_2_ gap (>6 mmHg) [198,199] Furthermore, measurement of ScvO_2_, a surrogate for global tissue oxygenation, in the context of a pCO2gap, a surrogate for cardiac output (or flow), may provide a more useful assessment of the effectiveness of a resuscitation. Thus, hemorrhagic shock should show ↓↓ScvO_2_ with ↑pCO_2_ gap associated with an ↑↑O_2_ER and ↑lactate. Distributive shock would appear essentially the same except for ↑ScvO_2_ and ↓pCO_2_gap [200].

## 5. Conclusions

This review examined first, the pathophysiology of hemorrhagic shock and second, current management strategies for acute blood loss in the polytrauma patient. Hemorrhagic shock is in part characterized by a critical reduction in global oxygen delivery (DO_2_) caused by an acute loss of O_2_ carrying capacity such that DO_2_ < DO_2 CRIT_ and O_2_ consumption becomes flow-dependent. During reductions in DO_2_ that do not fall below DO_2 CRIT_, oxygen availability is maintained by increased extraction of oxygen from blood delivered to the periphery. This compensatory mechanism can create a deceptive appearance of stability in the polytrauma patient who may have suffered a significant blood loss and is continuing to hemorrhage.

In addition, serious injury and hemorrhage are associated with one or more defects in coagulation, fibrinolysis, or both, which, predictably, exacerbate bleeding. Disorders of hemostasis are detectable in approximately 25–56% of polytrauma patients prior to initiating resuscitation [201]. Clot formation, centered on the generation of thrombin and fibrinolysis, centered on the generation of plasmin are two highly complex integrated plasma-based systems consisting of several proteins and endogenous inhibitors, which interact with the vascular endothelium under normal conditions and the endothelium and sub-endothelium at sites of injury. Acquired defects of hemostasis in the polytrauma patient include systemic damage to the endothelium (referred to as endotheliopathy), qualitative platelet defects (especially in patients with head injury), fibrinogen depletion, vWF dysfunction, or disorders of fibrinolysis, including fulminant hyperfibrinolysis and fibrinolytic shutdown. We have referred to these collectively as trauma-induced coagulopathy (TIC), which may manifest as either a hypocoagulable or hypercoagulabe (thrombotic) states. Precise descriptions of mechanisms responsible for most of these acquired hemostatic defects are incomplete. However, the excess mortality associated with TIC perhaps justifies close familiarity with fundamental precepts of hemorrhagic shock.

Furthermore, we have examined different aspects of DCR for the management of hemorrhagic shock in the polytrauma patient. During the last 70 years, the resuscitation of hemorrhagic shock has evolved from transfusion of whole blood; to transfusion of packed RBCs with large volumes of isotonic crystalloid; to balanced transfusion of RBCs, plasma and platelets, without crystalloid, and with or without fibrinogen replacement by transfusion of cryoprecipitate; to again transfusion of whole blood. At the same time, extension of viscoelastic assays from assessment of coagulopathies secondary to liver disease to polytrauma patients with TIC has been the basis for so-called goal-directed DCR strategies, which also include viscoelastic assessments of fibrinolysis not routinely included in conventional tests of coagulation. Acquisition of viscoelastic data (by either thromboelastography or rotational thromboelastometry) presents additional challenges during DCR, as trained personnel to perform the assay and experienced clinicians to accurately interpret the results are required.

We have described DCR in general terms as a structured approach to major trauma that integrates the principles of hemodynamic resuscitation, including massive transfusion to restore adequate O_2_ delivery, hemostatic resuscitation to treat or prevent TIC, and homeostatic resuscitation to treat or prevent extreme hypothermia, divalent cation imbalances, acidosis, and anticipated left-shifts in the OHD curve. Another important component of DCR is permissive hypotension which is predicated on perceiving hemorrhagic shock as a blood flow problem and not necessarily a blood pressure problem.

We believe future DCR protocols will demonstrate the increasing use of specific concentrates such as fibrinogen concentrate, or PCCs adapted specifically to the trauma patient in place of allogenic blood components. We predict that the most successful concentrate will likely be a combination of certain concentrates that individually have minimal impact on outcomes, but synergistically improve mortality significantly, for example, a fibrinogen concentrate combined with a factor XIII concentrate.

Finally, we expect that a more complete pathophysiologic description of hemorrhagic shock will require a substantially better understanding of microcirculation function [202] and hemorrhagic shock-induced disruption of the critical role the microcirculation holds in regulation of tissue perfusion. Moreover, we anticipate that specific treatments of disorders of the microcirculation in patients in hemorrhagic shock will mean significant modification of current practices of DCR.

## Abbreviations

DO_2_—Oxygen delivery, VO_2_—oxygen consumption, OHD—oxyhemoglobin dissociation curve, Hgb—hemoglobin, O_2_ER—oxygen extraction ratio, TIC—trauma-induced coagulopath, PAI-1—plasminogen activator inhibitor, TAFI—thrombin-activatable fibrinolysis inhibitor, DAMP—damage-associated molecular pattern, PAMP—pathogen-associated molecular pattern, HF—hyperfibrinolysis, FS—fibrinolysis shutdown, Ang-1,-2—anngiopoietin-1,-2, EC—endothelial cell, TM—thrombomodulin, MT—massive transfusion, LTOWB—low titer, type O, whole blood, DCR—damage control resuscitation.
